# Ten simple rules for good leadership

**DOI:** 10.1371/journal.pcbi.1010133

**Published:** 2022-06-09

**Authors:** Philip E. Bourne

**Affiliations:** School of Data Science and Department of Biomedical Engineering, University of Virginia, Charlottesville, Virginia, United States of America; Dassault Systemes BIOVIA, UNITED STATES

Over the years, I have had the opportunity to work directly with a number of leaders in science. I define these as people who lead their own laboratories, departments, and schools within universities, universities themselves, national laboratories, and funding agencies, like the National Institutes of Health (NIH), hereafter referred to as organizations. Some are distinguished scientists with Nobel Prizes, National Medals, and members of various academies. What makes some of them good leaders? My answer is personal and subjective. Note the “some of them” reference. Being a good scientist does not necessarily equate to good leadership, although there is some correlation, particularly in an era when science is a team sport requiring multiple types of expertise that needs to be led. Likewise, good leadership does not necessarily need to reflect good management skills. You can have thought leaders, but those leaders need to be able to delegate management tasks.

In broad terms, I would classify leaders of 2 types, enabling and autocratic. Surprisingly, both can be successful as regards the success of the organizations they lead, but autocrats make it a much less pleasant working environment for those who do the work of the organization, whether it be a lab or a larger organization. What follows are the rules for being an enabling leader who believes what is best for the individual is best for the organization. For each rule, I have provided an example from my own observations that exemplifies that rule.

## Rule 1: Lead by example

You cannot expect of others what you would not expect of yourself. If you expect others to work long hours, you need to work longer. If you expect others to meet deadlines, you need to meet them yourself, and so on. Leading by example leads to being respected for what you do on behalf of the organization. Being respected is a must for good leadership and it is not given, but earned over time. A leader I respected came to stay at our house having given a lecture the night before at my university to great acclaim. That day they were to have a fireside chat with me in front of a live audience. Were they sleeping in basking in the events of the night before? No, they were up before dawn doing their days work prior to this extra activity. Observing such dedication obviously sticks with one.

## Rule 2: Be humble

A great organization is facilitated by a good leader, but not solely the result of one. Recognize that and be humble. If you are building a great organization, there needs to be people who are smarter than you and better at doing certain things than you are. You can’t be the best at everything. Recognize this while rewarding and celebrating others for the skills they bring to the organization. Your team will respect you for it. Give credit whenever possible and take little for yourself. Say “we” instead of “I” as often as possible. Credit will come to you anyway if you are humble and leading an organization well. A Nobel Laureate I know, even though they are supporting the science, will not put their name on the paper unless they feel they have made a significant hands-on scientific contribution to the work at hand, being both humble and showing integrity.

## Rule 3: Be inclusive, equitable, and personal

We all like to be called out or otherwise recognized. You do, I do, so why wouldn’t others? When calling people out, it’s important to accurately recognize their contributions and be sure to include all who contributed. However, don’t be frivolous with such praise, which will dilute its value.

Inclusion starts with recognizing the individual and that begins with their name. I have seen great leaders be introduced to 20 people around the room and remember their names without writing them down. Some of us don’t have that ability, but addressing them by their name, even if you need to write it down and then look it up, speaks to the value of an individual. Beyond names, it’s knowing people for who they are, not just for what they do for the organization. The personal/human touch is very important. As far as possible, get to know all you lead as colleagues with personalities, families, and interesting backgrounds. Be unbiased. Treat all team members equally in all aspects of operating the organization.

## Rule 4: Lead by consensus and shared governance

Autocrats tend to lead by fiat leaving others to feel worthless or, at best, not fully appreciated. Enabling leaders seek opinions from a broad swath of people within the organization and act by consensus, or at the very least, explain in an open and transparent way why they are making a decision that goes against the consensus. A renowned leader I know would be sure they had the views of all the interviewees when they met with the job candidate at the end of the day. That input would immediately figure into the calculation of whether to hire this person. I was one such person that was hired that way.

## Rule 5: Be caring

People define an organization. Good leaders care for their people. It follows that the best organizations attract the best people—the word gets around—and can retain the best people. Caring means being compassionate, listening to those who look to you for leadership, and being empathic regarding family situations and other disruptions. The Coronavirus Disease 2019 (COVID-19) has amplified the importance of this approach.

Caring pays off not just for the organization, but for the leader. My own personal example is when a former student, now a professor, was on the search committee for a leadership position where I was a candidate. Our prior relationship did not influence his support—I had mentored him well—but presumably knowing of my caring first hand supported that important element of my value to the organization.

## Rule 6: Be visionary

Those in the organization will look to you to lead. Your vision will go a long way in defining the organization. If done right, it will appear as a collective vision. Everyone in the organization will feel that they are part of creating the vision. In a way, your job is to seed the vision and enable others to refine, nurture, and promote the vision as their own. Strategic plans have a role to play here. They offer a roadmap for all to follow. You will be successful when others speak of your vision as their own and you are proud of them doing so. Adoption is the highest compliment.

When our new university president and provost were appointed, they set about developing a strategic plan that took enormous efforts through surveys, town halls, and other forums to capture the views of all stakeholders (see Rule 3). That so-called “2030 plan” was then embodied in a single statement, “Be Both Great and Good.” Many stakeholders, including myself, use the phrase frequently. A simple statement of their vision shared by all in the organization.

## Rule 7: Be decisive

Some leadership decisions are hard. There is not always a clear decision, and people and the organization will be impacted. An element of risk-taking is then necessary. Decisions should not be made based on how people in the organization will react to them, but on what you believe is right for the organization. What is worse is to not make a decision and leave the organization in an indecisive state. Good leaders will make less than optimal decisions on occasion. The important thing to remember is to be transparent with all stakeholders, explaining why decisions were made and to work together toward a successful outcome. It is also important to acknowledge when you make a wrong decision and correct the mistake in a timely way in consultation with your team members.

An example I have seen a number of times occurs in hiring. The search committee and other stakeholders are split between who to hire and the leader has to make the decision. Being less than decisive may mean losing both candidates to other jobs, whereas making a decision will upset half your stakeholders. A good leader takes all features of both candidates into consideration as well of input from all stakeholders and makes very clear why the choice is being made and expects all to respect the choice when the candidate they did not choose comes on board.

## Rule 8: Get the right people on the bus and let them define the route

This is arguably the most important rule and implies you should pay close attention to who you hire. As I mentioned above, people define the organization. Hiring the right people for your organization is everything. In small organizations, one person who is a bad fit can upset the entire organization. Science thrives on innovation and so do organizations. Give your team members room to innovate. Don’t be over prescriptive as this will hobble smart people. Also, be adaptive yourself. The directions you take may not be what you originally planned. The smartest people with the best credentials may not be the best hires if they don’t have the personality or hunger to help the organization thrive. This is a fine balance in a scientific organization where the work of individuals is important. It may take mistakes to get it right.

As stated, the right people will be smarter than you are. The autocratic leader will find it hard to hire such people, which leads to suboptimal organizations where the best and brightest have been turned away.

A great leader I worked for did not give me the position I wanted and I moved on. However, I respected them for that decision, even initially, because they obeyed all the rules here. Over time observing the person that did get the job, I realized I would not have been the best fit, which would have left me and the organization not performing at our best.

## Rule 9: Delegate

You can’t do everything yourself. As an organization grows, this becomes more and more apparent. You need to surround yourself with a manageable number of direct reports that you fully trust to do the work of the organization. This applies even at the level of a small individual lab when it grows and the more senior, experienced members take on a leadership role.

In a larger organization, such as a university or funding agency, the dean will have a small number of associate deans as direct reports, and the director will have a small number of deputy directors, respectively. In highly functional organizations, these deputies are trusted and respected by the leader and have different skill sets, which, when taken together, enrich the whole enterprise.

## Rule 10: Enjoy the experience but know when to stop

Contentment with the role you play in the organization is contagious. If you are not inspired by what you are doing, how can others be? If you are not enjoying being a leader, either you are not cut out for the job or you are not leading the right kind of organization. You need to ask yourselves these difficult questions. There may also be a temporal nature to your happiness. Leading a small organization is different from building a small organization into a large one and leading that. Know when to come and when to leave.

I had the good fortune to watch a great leader build a new school within a great university prior to undertaking such an endeavor myself. That leader obeyed these 10 rules, but also knew when it was time to step aside. In doing so, they had built a team to continue, and they had a succession plan that meant the organization kept moving forward after they left. Hopefully, I have learnt that lesson as well.

There you have it. It is never too early to start thinking about leadership. There are endless books, videos, and courses on leadership. They may offer you better advice on being a leader. The most influential book I have read was “From Good to Great” by Jim Collins. Let me reiterate, that for me, most of what I have learnt about leadership and embodied in these rules was gained by observing leaders who I came to respect for what they have done for organizations. I acknowledge a few of them below. I like to think they have served me well in my own quest to be a good leader.

